# Vision-Based Autonomous Crack Detection of Concrete Structures Using a Fully Convolutional Encoder–Decoder Network

**DOI:** 10.3390/s19194251

**Published:** 2019-09-30

**Authors:** M. M. Manjurul Islam, Jong-Myon Kim

**Affiliations:** School of Electrical, Electronics and Computer Engineering, University of Ulsan, Ulsan 44610, Korea; m.m.manjurul@gmail.com

**Keywords:** structural health monitoring, image processing, computer vision, deep learning, concrete structure crack detection, visual geometry group network, semantic segmentation

## Abstract

The visual inspection of massive civil infrastructure is a common trend for maintaining its reliability and structural health. However, this procedure, which uses human inspectors, requires long inspection times and relies on the subjective and empirical knowledge of the inspectors. To address these limitations, a machine vision-based autonomous crack detection method is proposed using a deep convolutional neural network (DCNN) technique. It consists of a fully convolutional neural network (FCN) with an encoder and decoder framework for semantic segmentation, which performs pixel-wise classification to accurately detect cracks. The main idea is to capture the global context of a scene and determine whether cracks are in the image while also providing a reduced and essential picture of the crack locations. The visual geometry group network (VGGNet), a variant of the DCCN, is employed as a backbone in the proposed FCN for end-to-end training. The efficacy of the proposed FCN method is tested on a publicly available benchmark dataset of concrete crack images. The experimental results indicate that the proposed method is highly effective for concrete crack classification, obtaining scores of approximately 92% for both the recall and F1 average.

## 1. Introduction

Reliability, performance, and life cycle costs are real concerns for almost all in-service massive structures, such as buildings, bridges, nuclear facilities, hydroelectric structures, and dams. Cracks on these structures are a common phenomenon associated with various internal and external forces, including the corrosion of embedded reinforcement, chemical deterioration of concrete, and the application of adverse loading to the structure [1,2,3]. The appearance of cracks very often indicates significant distress within the structures. Therefore, to ensure the structural reliability and performance of the structure throughout its life, structural health monitoring (SHM) systems are needed to prevent catastrophic failure in the early stages [4,5,6]. SHM is the process of implementing a crack detection and characterization strategy for engineering structures.

In comparison to the traditional manual inspection-based crack detection system, computer vision and machine learning-based approaches are quickly becoming an integral part of the modern SHM of civil infrastructures to automate crack detection and identification systems [1,7,8]. These methods are mainly built upon common image processing techniques, such as segmentation, fuzzy clustering [9], pattern recognition, image filtering [10], histogram analysis [8,11], edge detection [12], and texture matching. Researchers in [12] applied various edge detection algorithms and found that the wavelet method is the most reliable among such approaches for the purpose of a crack detection system. Prasanna et al. [8] developed a histogram-based method for informative feature extraction, and then fuzzy logic was applied to the features for discussion. The outcomes of this algorithm on a real bridge highlighted the need for improvement in accuracy. Likewise, researchers performed clustering analysis to detect cracks using Canny and k-means clustering techniques [13].

To process a large volume of concrete structure image data regarding cracks, the machine learning-based classification approach has recently received significant attention [14,15]. In [16,17], the support vector machine (SVM) was applied to detect “crack” and “no-crack” conditions from concrete image data through extracting handcrafted manual features. The feature extraction process acts as a vital bridge between the raw image and rich feature vectors regarding cracks, which are used for classification. As the original feature vectors are significantly large, a dimension reduction method using principal component analysis (PCA) was employed to provide confined but informative feature components [18]. These components were tested with an SVM classifier, which showed a significant performance improvement. Other classifier techniques, such as an artificial neural network (ANN) [19], k-nearest neighborhood (k-NN), and a fusion of classifiers, e.g., fuzzy logic with SVM and a generic algorithm-support vector machine (GA-SVM), were applied to achieve higher accuracy in crack identification. 

In addition to the above classical machine learning approaches, deep learning techniques have recently achieved huge successes in pattern recognition, including in image classification, object detection, and computer vision. These deep learning approaches are successful because of their ability to handle and control a vast amount of big data and to automate the feature extraction and classification process, which inspire their applications to concrete structure crack detection problems. Zhang et al. [20] developed a deep convolutional neural network (CNN), which was directly applied to raw crack images to automate feature extraction and classification. This algorithm showed a superior performance compared to handcrafted methods. A computer vision-based method using a 256 × 256 × 3 CNN classifier integrated with sliding-window techniques was applied for concrete crack classification [11]. A pre-trained CNN with transfer learning [21] and a faster region-based CNN [22] have also been successfully applied to analyze concrete and detect steel and bolt corrosion. Authors in [23] used a convolutional neural network with an improved pooling technique such as Atrous Spatial Pyramid Pooling (ASPP) for concrete bridge crack detection. The purpose of ASPP is to replace the max-pooling to improve the detection accuracy. Most previous studies that have proposed crack detection methods based on image classification and/or object detection using boundary-boxes have performances that necessarily depend on manual parameters, such as size, length, and location information of the cracks. However, a concrete crack generally reveals itself as thin dark lines with varying directions and angles. Although boundary-box-based techniques showed a reasonable success in the case of crack detection, these methods are unable to provide accurate information about the crack path and density. Thus, pixel-wise image classification is desirable using sematic segmentation analysis, which acts to distinguish between “crack” and “no-crack” pixels in the image. Therefore, we deploy semantic segmentation to obtain more precise information about the crack path and density for the purpose of accurate crack detection. Semantic segmentation is a process of end-to-end learning that classifies an object on a pixel-by-pixel basis. The main idea is to capture the global context of a scene and provide information about the content of the image while also providing only the essential information about the locations of that content. Substantial literature regarding segmentation analysis can be found related to self-driving cars, medical imaging analysis, and the classification of terrain visible in satellite imagery [24,25]. Recently, Zhang et al. [26] used a fully convolutional network with a residual network as a backbone for concrete surface crack detection. Inspired by the recent result of semantic segmentation for precise object detection, we propose a fully convolutional network (FCN) with an encoder (forward/interface) and decoder (backward/learner) for accurate concrete crack detection. We use the visual geometry group network (VGGNet), a variant of the CNN, as a backbone for end-to-end training [27,28]. The proposed FCN with an encoder and decoder framework is applied not only to improve the detection accuracy but also to perform semantic analysis through backward learning to capture context of the crack such as the path and trend. The performance of the proposed FCN is verified with an open-source dataset [29] of concrete crack images.

The remainder of this paper is organized as follows. In Section 2, the overall methodology is presented, including the proposed FCN model. The experimental results, with robustness and reliability analysis using numerous evaluation matrices, are presented in Section 3. Finally, we conclude the paper in Section 4.

## 2. Materials and Methods

**Figure 1:**
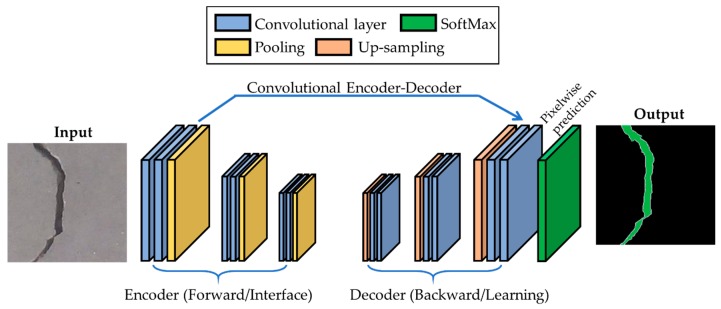
Block diagram of the proposed fully convolutional network (FCN) with the encoder–decoder framework.

Due to the complexity of trends of concrete crack propagation, boundary box-based classification and detection algorithms are unable to properly identify cracks. Compared to boundary box-based classification and detection tasks, semantic segmentation is the process of end-to-end learning, which classifies each pixel of the input image. As concrete crack detection is a problem of segmentation analysis, we develop an encoder–decoder fully convolutional network (FCN) for the task of segmenting an input image of a concrete crack into no-crack and crack pixels for detecting a crack. The proposed FCN model is composed of an encoder framework and corresponding decoder, which is illustrated in Figure 1. 

### 2.1. Proposed FCN Architecture for Crack Classification

In Figure 1, we can see that the proposed FCN is a fully convoluted network in which the encoder–decoder frameworks act as a backbone to perform end-to-end training. 

#### 2.1.1. Encoder Network

**Figure 2:**
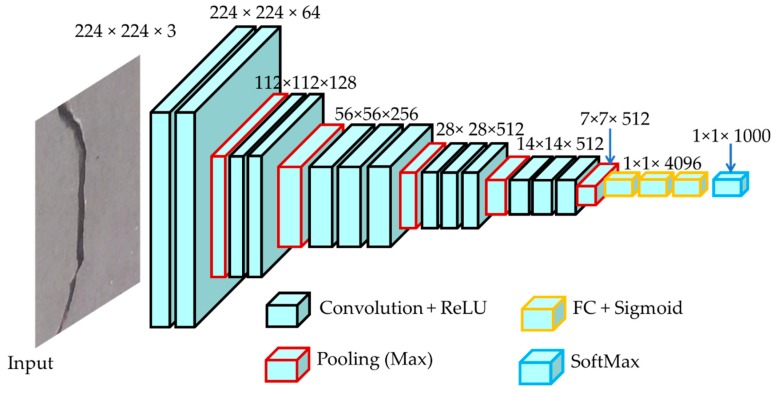
The proposed encoder framework using the visual geometry group network (VGGNet) architecture with some modification. In the figure, ReLU stands for the rectified linear unit, and FC defines the fully connected network.

An encoder is an algorithm that extracts abstract features containing all necessary information about the input to perform the correct segmentation, detection, and classification. The encoder mainly consists of the convolution and pooling layers of a classification network. Modern classification networks can be used as an encoder in the FCN. In the paper, we use the visual geometry group network (VGGNet) [28]. The biggest success of this network is that the depth of the network is high, which is important to ensure good performance. VGGNet is a very deep convolutional network and is the most widely used pre-training convolution architecture for the ImageNet dataset. The encoder in the proposed FCN is based on VGGNet with some modification, which consists of 13 convolutional layers containing 3 × 3 filters, and all pooling is 2 × 2. In this structure, the convolutional layer is simply followed by a pooling layer, and finally, a fully connected layer. A typical encoder of our proposed FCN is illustrated in Figure 2.

A convolution is a mathematical operation acting upon two sets of information, which performs addition, integration, multiplication, or a derivative. The convolution used here is as follows:

(1) $$y=x*w\Rightarrow y\left[i\right]=\overset{+\propto }{\underset{j=-\propto }{\sum }}x\left[i-j\right]w\left[j\right].$$

The two sets of information are the input data, x, and a convolution filter, which is also called the kernel, w. The convolutional operation is performed by sliding the kernel over the entire input, which produces a feature map. In practice, different filters can be utilized to perform multiple convolutions to produce distinct feature maps. These feature maps are finally integrated to formulate the final output from the convolution layer. 

Activation functions are used after the convolution operation to introduce nonlinearity to the model. Various activation functions such as a linear function, sigmoid, or tanh can be used, but the rectified linear unit (ReLU) is used in the proposed VGGNet, as it can train the model much faster and ensure near-global weight optimization in contrast to other activation functions. The ReLU activation function is defined as follows:

(2) $$f\left(x_{i}\right)=max\left(0,x_{i}\right).$$

The pooling layer appears next to the convolution layer. This layer down-samples each feature map to reduce its dimension, which, in turn, reduces overfitting and the training time. The max-pooling approach is used in the proposed VGGNet, which simply selects the maximum value in the pooling window.

The FC layer is essentially a fully connected layer of the artificial neural network [30]. In a nutshell, the VGGNet, convolution, and pooling layers automatically extract low-level features such as edges and lines for cracks, and the FC layer performs the classification task, which is based on these low-level features. The activation function used in this final classification layer is the SoftMax function, which assigns a probability value to each class such that the probabilities add up to 1. The SoftMax function, which could be replaced by a similar activation function, is defined as

(3) $$S\left(y=j\vee \phi^{\left(i\right)}\right)=\frac{e^{\phi^{\left(i\right)}}}{\sum_{j=0}^{k}e^{\phi_{k}^{\left(i\right)}}}.$$

If the weight matrix is denoted as *W* and the feature matrix by *X*, then ϕ in the above equation is generalized as

(4) $$\phi =\overset{k}{\underset{i=0}{\sum }}W_{i}X_{i}=W^{T}X.$$

#### 2.1.2. Decoder Network

**Figure 3:**
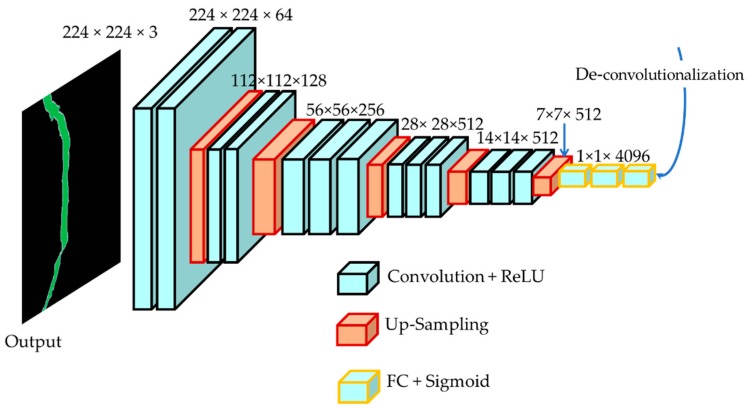
The proposed decoder framework using VGGNet architecture.

In the encoding phase, the process mainly automatically extracts very low-level features and performs classification. For decoding, we also use the same VGGNet architecture for consistency, as shown in Figure 3. The decoder framework applies deconvolution and up-sampling of the layers to reconstruct the corresponding segmented high-level image from the low-level features. As shown by the feature generated by the encoder, a 1 × 1 convolution is applied to create a low-resolution segmentation. Then, the output is up-sampled by subsequent deconvolution layers to generate high-resolution features. 

**Figure 4:**
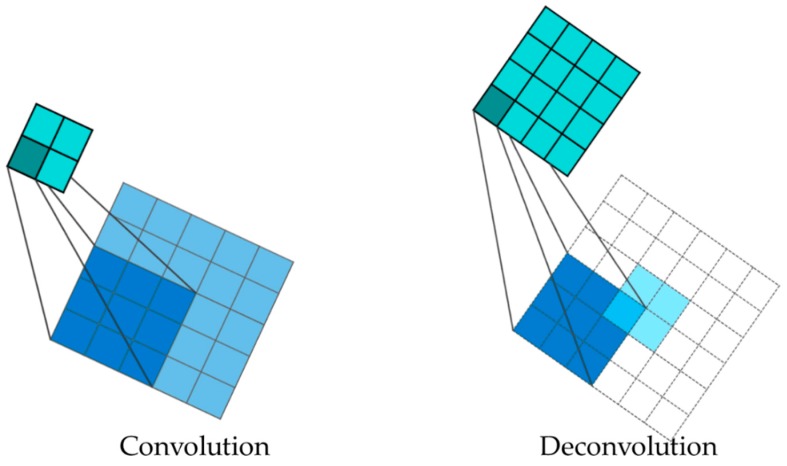
Process of convolution (**left**) and deconvolution (**right**) in proposed FCN.

Due to the nature of the convolution process in the encoder, the output size is smaller than the input size (Figure 4 (left)). This is an issue for the decoding process. Thus, in this paper, the output size is increased through a method called deconvolution and up-sampling (Figure 4 (right)) to make the input look the same as the image (to increase the spatial information). 

**Figure 5:**
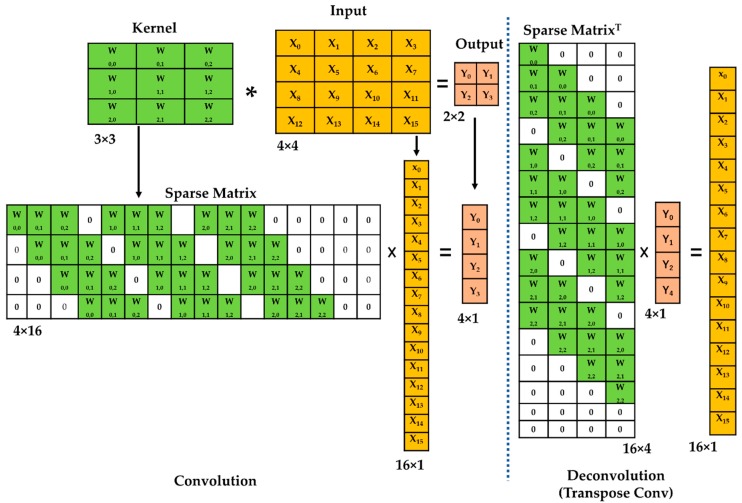
An example of the deconvolution process using transpose convolution. In the figure, *—Convolution; **×**—Matrix Multiplication.

Deconvolution, in other words, is called the transpose convolution [24]. Figure 5 shows the process of traditional convolution operations (Figure 5 (left)) and transpose convolution (Figure 5 (right)). To explain the transpose convolution process, the sparse matrix of the kernel is transposed and multiplied with the output. Therefore, the input size is increased to resolve the loss of spatial information in the decoding process when the output is downscaled due to convolution characteristics.

### 2.2. Concrete Crack Image Dataset

In this paper, we used an open-source dataset of concrete crack images from various campus buildings of the Middle East Technical University [27] for classification and segmentation. This dataset consists of 40,000 concrete surface images with 224 × 224 pixels with RGB channels, which are equally split into “crack” and “no-crack” classes. High-resolution images have variance in terms of the surface finish and illumination conditions. No data augmentation in terms of random rotation or flipping is applied. A detailed description of the dataset is given in Table 1.

## 3. Results and Discussion

In this section, we verify the efficacy of the proposed FCN method using the open-source concrete crack images dataset (see Table 1) for classifying cracks. The model performance is evaluated using several performance metrics. They are briefly described in Section 3.1.

### 3.1. Performance Metrics

#### 3.1.1. Structural Accuracy

**Figure 6:**
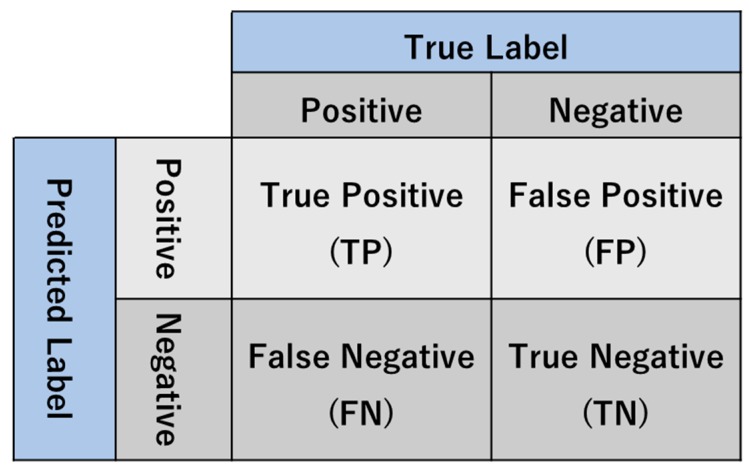
The confusion matrix.

The structural accuracy (SA) metric is mainly designed for examining the performance of a classification problem. SA is a single number that is extracted from the parameters of a confusion matrix. Figure 6 depicts a 2 × 2 confusion matrix, and several basic concepts are given as follows:

(1) TP defines the number of positive observations predicted to be positive. In our model, TP represents the number of cracks that are correctly classified as a crack.
(2) TN defines the number of negative observations predicted to be negative. In our model, TN represents the number of backgrounds that are correctly classified as background.
(3) FP defines the number of negative observations predicted to be positive. In our model, FP represents the number of backgrounds that are incorrectly identified as a crack.
(4) FN defines the number of positive observations predicted to be negative. In our model, FN represents the number of cracks that are incorrectly identified as background.

Therefore, SA is calculated for a binary classification task as follows:

(5) $$SA=\frac{TP+TN}{TP+TN+FP+FN}.$$

#### 3.1.2. F1-Score

The F1-score is a useful measure compared to the SA in cases where the dataset has a different class distribution. In such a scenario, a high SA does not imply a robust model. The F1 score is the harmonic mean of precession, P, and recall, R, and defined as follows:

(6) $$F1-score=2\times \frac{P*R}{P+R}.$$

Here, P and R are expressed as follows:

(7) $$P=\frac{TP}{TP+FP},$$

(8) $$R=\frac{TP}{TP+FN}.$$

As the F1 score involves both false positives and false negatives, this implies that the F1 score is a more useful measure than the SA for a dataset with different distributions.

### 3.2. Performance Analysis of Proposed FCN Model

**Figure 7:**
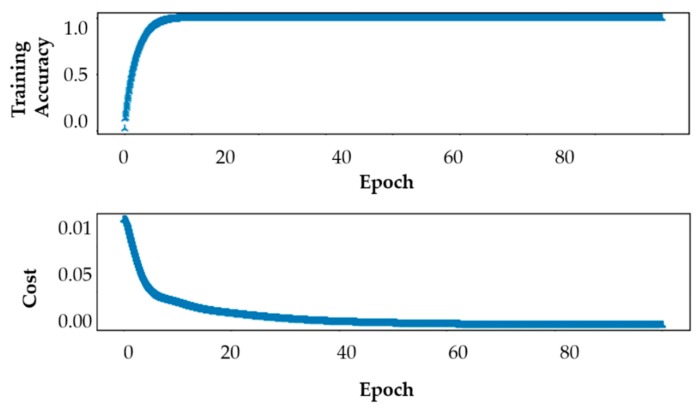
The accuracy (**top**) and cost function performance (**bottom**) of VGGNet during training.

For the proposed FCN model, it is important to report the performance cost function and accuracy of a VGGNet classifier during training to validate the performance of the proposed method. The classifier is trained for 100 epochs. Figure 7 presents the results of training accuracy and cost function performance against the number of epochs. From the results, it is reasonable to infer that as the number of epochs is increased, the cost function value starts to decrease until it becomes almost zero at the 60th epoch, while the training accuracy starts to increase until it becomes almost one at the 15th. Both training accuracy and cost function performance trends are important for good generalization performance.

We compared the performance of the proposed method with recent models such as the SVM [31] and CNN [32]. The SVM is trained using some of the widely featured elements. The cardinality of the feature vector is 85-dimensional and composed of a mean RGB value, hue histogram, local binary pattern, saturation histogram, and texton histogram [31,33]. These features are mainly based on the color and texture of images, which are related to the binary level that indicates the presence or absence of a crack. In [32], raw images are directly used with a CNN classifier to learn the discriminative features automatically. The results are presented in Table 2. According to the results, it can be seen that the proposed method achieves performances of 91.3%, 94.1%, 92.1%, and 92.8% for the precision, recall, F1-score, and SA, respectively.

The proposed method also significantly outperforms existing methods, yielding an average improvement of 10.93% from CNN and 20.93% from SVM in terms of the SA, as can be seen in Table 2.

As the SVM classifier’s performance depends on the handcrafted features, the local distortion and scaling information cannot be captured using manually extracted features. Although the CNN automates the feature learning and classification process, it still learns the same objects for all crack instances. As the concrete crack appears as a strip of line with varied angles and directions, this validates the use of the FCN model with encoder–decoder frameworks for crack detection.

**Figure 8:**
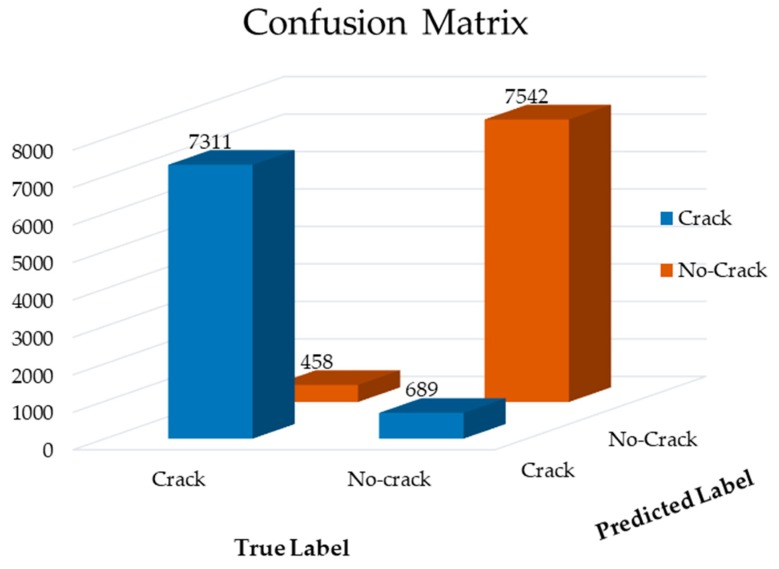
The confusion matrix of the proposed model.

Furthermore, to verify the performance of the proposed method, we provide the result of the confusion matrix. The confusion matrix is effective to visualize the classifier performance that shows the actual versus predicted accuracy. To measure the test performance, we provide 40% of “crack” and “non-crack” data (see Table 1). Figure 8 presents the results of a confusion matrix, which shows that correct-classification rates are significantly high in comparison to the miss-classification rate. In this result, out of 8000 crack samples, 689 samples are miss-classified as the non-crack class. 

**Figure 9:**
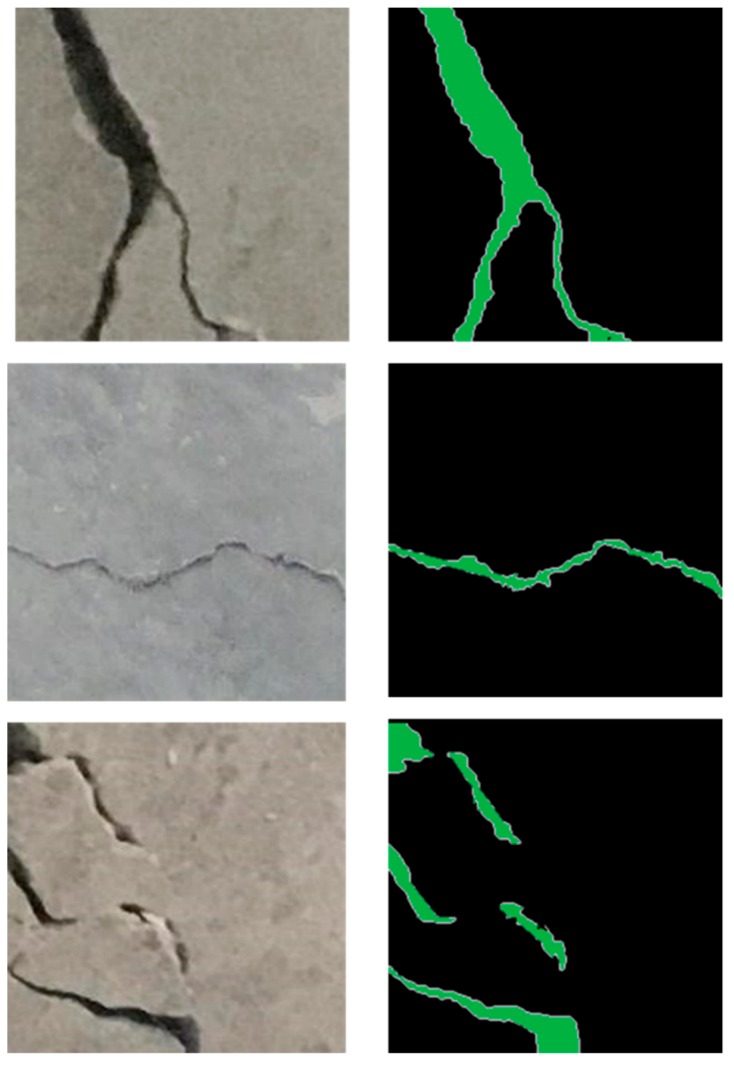
Examples of segmentation results for test images.

In addition to quantitative evaluation, to process a testing image for reconstruction, the proposed FCN model can provide each point centered within the image and the probability of being a crack or non-crack. This procedure yields a probability map. The probability of a point can be calculated by averaging the probability of each segment generated by randomly rotating it around its center pixel using the method proposed in [34]. Figure 9 displays the results of segmentation using the proposed FCN model for three different scenes. The pixels in green and black denote crack and no-crack, respectively. According to the results, it can be seen that the proposed model is highly effective at capturing all the features involving cracks in the images. 

## 4. Conclusions

In this paper, we developed a vision-based crack detection method through semantic segmentation analysis. The proposed FCN-based segmentation method was built upon encoder and decoder frameworks to perform end-to-end learning to classify each pixel. VGGNet, a benchmark network for segmentation, was used as a backbone for our FCN. This FCN is trained using an open-source concrete crack image dataset from campus buildings of the Middle East Technical University for model selection. Finally, the optimized FCN model was validated with unknown test data and yielded approximately 92% for both the accuracy and F1 scores. Furthermore, the proposed FCN model was highly accurate in identifying the crack path. Though the proposed FCN model showed significant success in terms of crack classification and trend detection, other backbone networks, such as AlexNet [35], InceptionV3 [36], and ResNet [37], can be used for model selection. 

## Figures and Tables

**Table 1:** Dataset description.

Number of Images	Size (Pixels)	Crack	No-Crack	Training	Validation	Test
40,000	224 × 224	20,000	20,000	40%	20%	40%

**Table 2:** A comparison of the support vector machine (SVM), convolutional neural network (CNN), and the proposed FCN model.

Methods	Precision	Recall	F1-Score	SA
SVM	68.75	73.33	70.96	71.87
CNN	88.75	78.02	83.04	81.87
Proposed FCN	91.3	94.1	92.7	92.8
